# Challenges in Photoinduced Electron Transfer Systems of Metal Complexes

**DOI:** 10.3390/mi17070799

**Published:** 2026-06-30

**Authors:** Yuki Murayama, Daisuke Nakane, Takashiro Akitsu

**Affiliations:** Department of Chemistry, Faculty of Science, Tokyo University of Science, 1-3 Kagurazaka, Shinjuku-ku, Tokyo 162-8601, Japan

**Keywords:** photoinduced electron transfer, photoredox, metal complexes, dye-sensitized solar cell (DSSC), reduction of carbon dioxide (CO_2_)

## Abstract

This review aims to clarify the molecular design principles and operational challenges of photoinduced electron transfer (PET) and photoredox processes in metal complexes. The manuscript is structured to include a survey of established conventional systems, such as Ru complexes, followed by our own research on cost-effective photosensitizers for dye-sensitized solar cells (DSSCs) and carbon dioxide (CO_2_) reduction. Crucially, our main conclusion emphasizes that achieving high optoelectronic efficiency requires the balanced optimization of excited-state lifetimes, orbital distributions, and matrix environments, rather than a simplistic “one-size-fits-all” approach. Finally, based on the fundamental principles of metal complexes and photocatalytic materials, we offer a critical analysis of the practical challenges and reasons behind our unsuccessful experimental outcomes. Thus, this study provides a perspective on unsuccessful molecular design, comparing typical examples.

## 1. Introduction

Photoinduced electron transfer (PET) in transition metal complexes is a cornerstone of modern photochemistry. They integrate light harvesting, charge separation, and redox reactivity within a single molecular platform. For decades, coordination chemists have leveraged metal polypyridyl complexes to elucidate how visible-light absorption generates electronically excited states with properties that diverge profoundly from their ground-state counterparts [1]. These fundamental mechanistic studies established the conceptual framework that now underpins the rapid expansion of photoredox catalysis, dye-sensitized solar energy conversion, and a diverse array of photocatalytic transformations [2]. Within this context, Ru(II) and Ir(III) polypyridyl complexes have emerged as uniquely versatile systems [3]. Their widespread utility stems directly from a rare combination of intense visible-light absorption, robust chemical stability, and highly accessible excited-state electron-transfer chemistry.

The fundamental driving force of photoredox chemistry lies in the light-induced generation of electronically excited states that possess enhanced thermodynamic oxidation and reduction potentials [4]. Upon absorbing a photon, a photocatalyst is promoted from its ground state to an excited state: a process that extends beyond a mere energetic transition to cause a profound redistribution of intramolecular electron density [5]. In transition metal complexes, this reactivity is predominantly governed by metal-to-ligand charge-transfer (MLCT) states, where an electron shifts from a metal-centered orbital to a ligand-centered orbital [6]. This directional electronic transition formally oxidizes the metal center while reducing the ligand, effectively yielding an intramolecularly charge-separated species. Because this localized charge separation stores substantial chemical potential in the excited state, the resulting complex functions simultaneously as a more potent oxidant and powerful reductant than its ground-state counterpart, thereby enabling light to drive otherwise thermodynamically forbidden chemical transformations.

Tris(2,2′-bipyridine)ruthenium(II), [Ru(bpy)_3_]^2+^, serves as the definitive benchmark in modern photochemistry, setting the gold standard against which newly developed photosensitizers are continually evaluated [7]. This complex holds such an expansive historical legacy that it has been aptly dubbed the “fruit fly of photophysics,” a testament to how profoundly it has shaped our fundamental understanding of excited-state processes in coordination chemistry [8]. The enduring utility of [Ru(bpy)_3_]^2+^ stems from a robust combination of synthetic accessibility, structural tunability via ligand modification, exceptional photostability under repeated excitation, and a strong emissive profile that facilitates detailed mechanistic interrogation. When dissolved in acetonitrile, its electronic profile features an intense ligand-centered band near 285 nm and prominent visible MLCT absorption at approximately 452 nm (1.3 × 10^4^ M^−1^cm^−1^) [9]. These photophysical characteristics ensure highly selective visible-light excitation, thereby avoiding unwanted direct photoexcitation of most organic substrates present in the reaction mixture.

**Figure 1:**
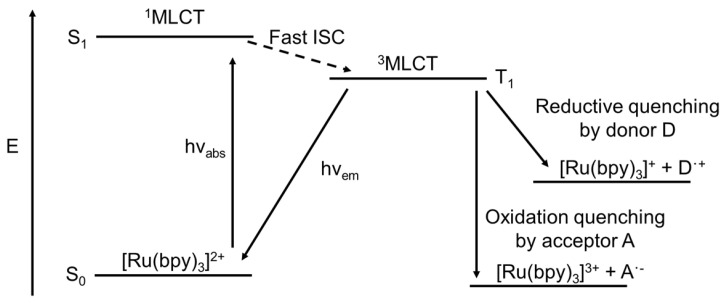
Typical Jablonski diagram of [Ru(bpy)_3_]^2+^. (1) Light absorption promotes [Ru(bpy)_3_]^2+^ to the transient ^1^MLCT state. (2) Rapid intersystem crossing converts this singlet state to the long-lived ^3^MLCT state with almost unit efficiency. (3) The ^3^MLCT state deactivates through characteristic phosphorescence or drives photocatalytic redox reactions.

A highly effective transition metal photocatalyst must simultaneously exhibit high quantum efficiency in reactive excited-state formation and a sufficiently long lifetime to engage in bimolecular chemistry prior to relaxation (Figure 1). This core principle of coordination photochemistry is epitomized by [Ru(bpy)_3_]^2+^; upon visible-light excitation, it initially accesses a singlet metal-to-ligand charge-transfer (^1^MLCT) manifold and undergoes ultrafast intersystem crossing to yield the emissive triplet MLCT (^3^MLCT) state [10]. This resulting excited state is remarkably long-lived on a molecular timescale. Its lifetime expands into hundreds of nanoseconds depending on the medium and ambient conditions used [11]. This durable lifetime provides an ample window for diffusion-controlled bimolecular quenching and productive electron or energy transfer (EnT) with external donors, acceptors, or semiconductor surfaces [11,12]. Furthermore, the distinct emissive characteristic of this ^3^MLCT state, featuring luminescence centered near 620 nm, has established [Ru(bpy)_3_]^2+^ as both an invaluable functional photocatalyst and an indispensable mechanistic probe for tracking electron-transfer kinetics [12].

The selection and optimization of photoredox-active metal complexes are ultimately governed by a delicate interplay of thermodynamics, kinetics, and excited-state dynamics, rather than a simplistic “one-size-fits-all” approach [13]. These fundamental parameters directly dictate the core design criteria for an effective photocatalyst: a large absorption cross-section in a transparent spectral window of the reaction medium, a high quantum yield of reactive excited-state formation, and a lifetime sufficient for diffusion-controlled electron transfer [14]. Furthermore, the complex must exhibit reversible photophysical and electrochemical properties, with ground- and excited-state redox potentials finely tuned to match the desired chemical transformation. For instance, cyclic voltammetry of the benchmark [Ru(bpy)_3_]^2+^ complex reveals a reversible metal-centered oxidation alongside ligand-centered reductions, establishing the precise electrochemical foundation of its MLCT reactivity [15]. More broadly, these observations emphasize that the driving force of a photoredox reaction cannot be inferred solely from the absorption maximum; rather, it depends entirely on how the absorbed photon energy reshapes both the oxidation and reduction potentials within the excited state.

The overall efficiency of photoredox catalysis hinges on a tight kinetic competition between productive forward chemistry of charge-separated intermediates and energy-wasting back electron transfer. Mechanistically, PET in these molecular systems proceeds via either oxidative or reductive quenching pathways. Upon photoexcitation to its excited state, the photocatalyst can either donate an electron to an acceptor—generating an oxidized complex—or accept an electron from a donor, yielding a reduced species. Crucially, preventing geminate or diffusional recombinations of these charge-separated intermediates is vital for both homogeneous photoredox networks and interfacial systems like semiconductor sensitization. Indeed, the exact excited-state dynamics that established [Ru(bpy)_3_]^2+^ as a paradigm for solution-phase electron-transfer kinetics also enabled its phosphonated or carboxylated derivatives to pioneer interfacial electron injection into TiO_2_ conduction bands in early DSSCs [16,17,18].

The architectural design of metal-based photosensitizers entails a fundamental kinetic trade-off between maximizing excited-state population efficiency and prolonging excited-state lifetimes [19]. In numerous transition metal platforms, robust spin–orbit coupling efficiently drives intersystem crossing toward triplet state formation, yet this same coupling often accelerates undesirable radiative and nonradiative deactivation pathways. Conversely, electronic states that exhibit weak coupling to the ground state can achieve extended lifetimes; however, they typically suffer from inefficient population rates [20]. To circumvent this inherent limitation, a prominent modern strategy couples an inorganic chromophore with organic donor or acceptor frameworks. This configuration ensures that initial photoexcitation and subsequent intersystem crossing proceed through MLCT gateway states, after which the triplet excitation is vectorially displaced away from the metal center into ligand-to-ligand or intraligand charge-transfer states [21]. These engineered trap states effectively preserve spatial charge separation while suppressing rapid back-decay, thereby expanding the available kinetic time window for downstream photocatalytic transformations.

Modern photocatalyst design has shifted from simple orbital or redox-potential tuning toward a comprehensive control of excited-state landscapes and inter-state electronic connectivity [22,23,24]. This paradigm shift highlights that the overall rate of PET is not determined by energetics alone. Indeed, contemporary theoretical analyses—including semiclassical Marcus treatments—emphasize the intertwined roles of thermodynamic driving force, reorganization energy, and electronic coupling between the respective donor and acceptor states. Systematic studies on Ru(II)-based donor-acceptor assemblies demonstrate that even when multiple charge-transfer pathways are thermodynamically accessible, the dominant cascade is frequently dictated by the specific magnitude of the electronic coupling matrix element. Consequently, whether an initial triplet MLCT state evolves into a long-lived ligand-to-ligand or intraligand charge-separated state depends not only on the relative energy levels of these states but also on how effectively they communicate electronically along the nuclear reaction coordinate.

The fundamental photophysical principles derived from [Ru(bpy)_3_]^2+^—such as excited-state charge separation, photoinduced redox-state inversion, lifetime engineering, and the control of electron-transfer pathways—continue to define the paradigm of modern coordination photochemistry [25,26,27]. This enduring conceptual framework explains why metal-complex photoelectron transfer remains a highly durable and evolving research theme. Pioneering spectroscopic studies and early electron-transfer quenching experiments established the MLCT nature of its emissive state, demonstrating that excited coordination compounds could engage in bona fide single-electron transfer with external molecular substrates. These historical breakthroughs laid the indispensable foundation for contemporary advances in photoredox catalysis, artificial photosynthesis, and molecular solar energy conversion. Consequently, even as current research increasingly transitions toward earth-abundant Fe, Co, or Cu alternatives to Ru, the benchmark Ru(II) complex remains the ultimate conceptual and practical yardstick for evaluating newly developed chromophores.

Transition metal complexes remain uniquely powerful platforms for solar-energy conversion because their orbital energies, ligand fields, redox properties, and intersystem crossing efficiencies can be systematically tailored through precise molecular design [28,29,30]. Conceived from this perspective, metal-complex PET is best understood not merely as an isolated reaction class, but as a unifying conceptual framework that illustrates how molecular architecture governs the transformation of light into chemical potential. The central challenge in this field lies in designing sophisticated complexes where photon absorption, excited-state formation, charge separation, and forward electron transfer all operate with high quantum efficiency, while simultaneously suppressing parasitic relaxation and charge–recombination pathways. Consequently, the rigorous study of PET in coordination systems continues to deliver both fundamental mechanistic insights and practical design principles for the development of next-generation photocatalysts, photosensitizers, and molecular solar-energy-conversion devices.

This review introduces photoelectron transfer in metal complexes, with a focus on the Ru complex described in Section 1 (Introduction). Section 2 discusses the positional relationship between the two metal ions (metal complexes) of the oxidizing and reducing species, as well as their application to carbon dioxide reduction catalysts using multiple catalytic complexes and dye-sensitized solar cells that receive light and supply electrons to semiconductors. Section 3 “verifies” the molecular design in Section 2 by examining a failed example, keeping dye-sensitized solar cells in mind. In Section 4, we conclude by discussing points of focus in computational chemistry for molecular design as a future outlook.

## 2. PET Using Metal Complexes

### 2.1. Dinuclear Complexes

**Figure 2:**
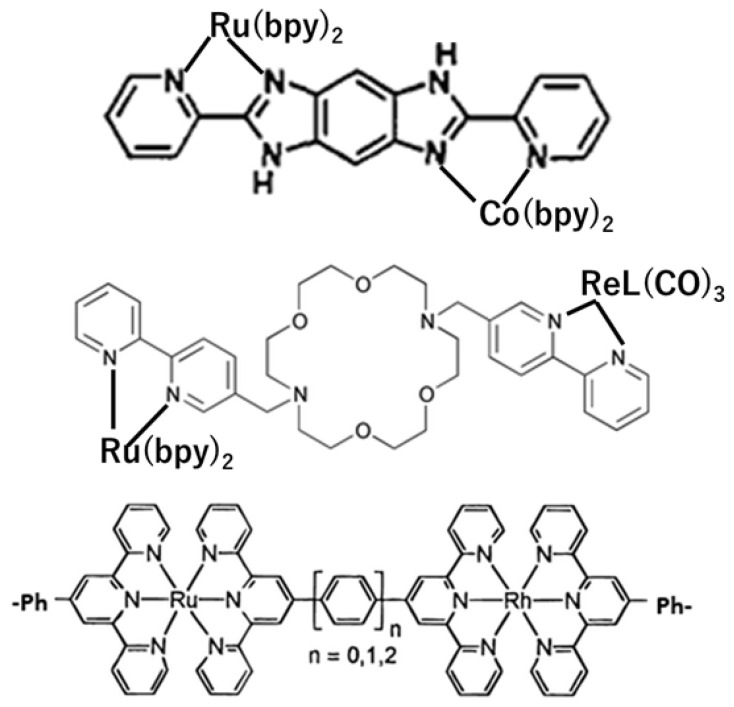
Examples of Ru-Co [31], Ru-Re [32], and Ru-Rh [33] dinuclear complexes. These studies investigate the mechanism of so-called inner-sphere electron transfer in dinuclear complexes. Here, redox-active metal centers—bearing ligands with extended conjugated systems (bpy or terpy ligands)—are linked by a bridging ligand that maintains a fixed distance between metals.

Since photoelectron transfer is also a redox reaction, it is fundamentally necessary for a complex that absorbs light and is oxidized, and a complex that accepts electrons and is reduced, to exist under appropriate conditions. By appropriately designing dinuclear complexes, it is possible to arrange two metal ions in a spatially desirable positional relationship. Here, we introduce examples of such metal complexes, including supramolecular systems (Figure 2).

Supramolecular photocatalyst design favors non-conjugated bridging ligands over fully conjugated networks to maintain high lowest unoccupied molecular orbital (LUMO) energy levels and preserve the strong reducing power of the catalytic center. Through this structural approach, Ru(II)-Re(I) dinuclear and tetranuclear complexes achieve significantly higher carbon dioxide (CO_2_) reduction activity and carbon monoxide (CO) selectivity than corresponding bimolecular mixtures [34].

Mechanistically, this efficient CO_2_ reduction is driven by a visible-light-induced electron-transfer cascade followed by a slow, thermodynamically neutral intramolecular relocation. Upon visible-light absorption, the selectively excited Ru triplet MLCT state undergoes reductive quenching by 1-benzyl-1,4-dihydronicotinamide to generate a one-electron-reduced species. Transient absorption spectroscopy verifies that the accepted electron initially localizes on the Ru bipyridine ligand before transferring to the rhenium site via an intramolecular pathway with a near-zero free-energy change for up to several seconds. This electronic translocation ultimately induces dissociation of chloride ligands from the rhenium center, creating a coordinatively unsaturated active site that facilitates rate-limiting CO_2_ reduction.

The significant kinetic disparity between ultrafast photoinduced forward electron transfer and slow back electron transfer in Ru(II)-Co(III) binuclear systems is primarily dictated by spin-state transitions and the resulting changes in internal reorganization energy. Yoshimura and co-workers [31] elucidated this mechanism by synthesizing three types of dinuclear complexes where the Ru(II) and Co(III) sites were linked via different bridging ligands. Upon generating the Ru(II) MLCT excited state via a 532 nm laser pulse, a sequential two-step forward electron transfer occurs: a low-spin Ru(III)-Co(II) intermediate with a negligible internal reorganization energy forms first, followed by rapid intersystem crossing of the Co(II) center from its low-spin doublet state to its high-spin quartet ground state. Conversely, Marcus’ theory analysis reveals that dark back electron transfer from the high-spin Co(II) to Co(III) is remarkably retarded because the significant accompanying change in coordination bond lengths imposes a massive internal reorganization energy of approximately 1.2 eV. In addition, a large negative entropy change (−1.3 meV/K) drastically diminishes the pre-exponential frequency factor.

Intramolecular electron transfer within Ru(II)-Re(I) carbon dioxide reduction photocatalysts occurs via a dominant through-bond super-exchange tunneling mechanism rather than a through-space pathway. Yamazaki and co-workers [35] demonstrated this behavior using sub-nanosecond time-resolved infrared spectroscopy to track a series of binuclear complexes linked by various alkyl or ether bridging chains. Following visible-light excitation of the Ru unit to its triplet MLCT state and subsequent rapid reductive quenching by 1,3-dimethyl-2-phenyl2,3-dihydro-1H-benzo[d]imidazole, the intramolecular electron transfer rate constant (*k*_ET_) for the rhenium catalytic center was directly monitored by tracking the low-energy shifts in the rhenium carbonyl infrared stretching bands. For complexes with a single flexible chain, the logarithm of *k*_ET_ exhibited a linear relationship against the total number of bonds along the bridging chain, yielding an attenuation coefficient of 0.74 per angstrom. This characteristic tunneling behavior, combined with the substantial acceleration rate observed in a dual-ethylene-chained complex, strongly supports a through-bond pathway mediated by the bridge’s electronic states.

**Figure 3:**
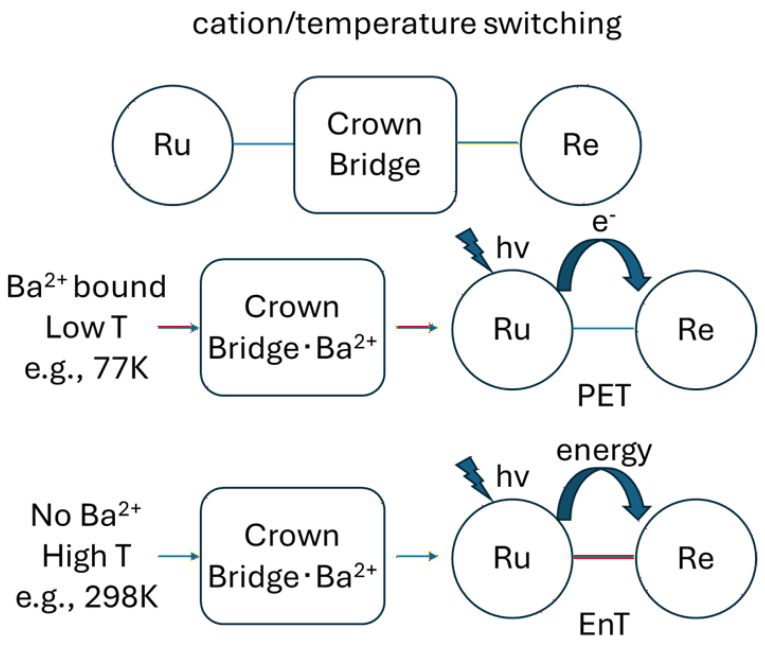
Photochemical pathway switching in a Ru(II)–Re(I) heterobinuclear assembly linked by a diaza-18-crown-6 macrocycle [32]. At room temperature, rapid PET from the crown ether nitrogen quenches the Re-based emission, which is effectively blocked upon Ba^2+^ encapsulation. In a 77 K frozen matrix, PET is thermally suppressed. As a result, Re-to-Ru EnT becomes the dominant pathway. The addition of Ba^2+^ at 77 K further retards this EnT rate by approximately thirty-fold due to electrostatic repulsion-driven structural expansion.

The innovative integration of a macrocyclic spacer into a Ru(II)-Re(I) heterobinuclear assembly enables dynamic switching in four distinct photochemical pathways controlled by temperature and metal-ion coordination. Encinas and co-workers engineered this system using a diaza-18-crown-6 macrocycle to link the Ru(II) and Re (I) luminescent units [32]. In ambient fluid solutions without barium ions, rapid PET from the amine nitrogen of the crown ring to the excited rhenium site (rate constant of 1.2 × 10^10^ s^−1^) completely quenches the rhenium-based emission. Barium ion encapsulation blocks this pathway by shifting the oxidation potential of nitrogen atoms. Conversely, in a frozen matrix at 77 K where the electron-transfer intermediates are thermodynamically destabilized, only Re-to-Ru EnT operates (rate constant of 2 × 10^8^ s^−1^). Ba binding at 77 K further retards this EnT rate by roughly thirty-fold (7 × 10^6^ s^−1^) due to structural expansion driven by electrostatic repulsion between the metal centers (Figure 3).

**Figure 4:**
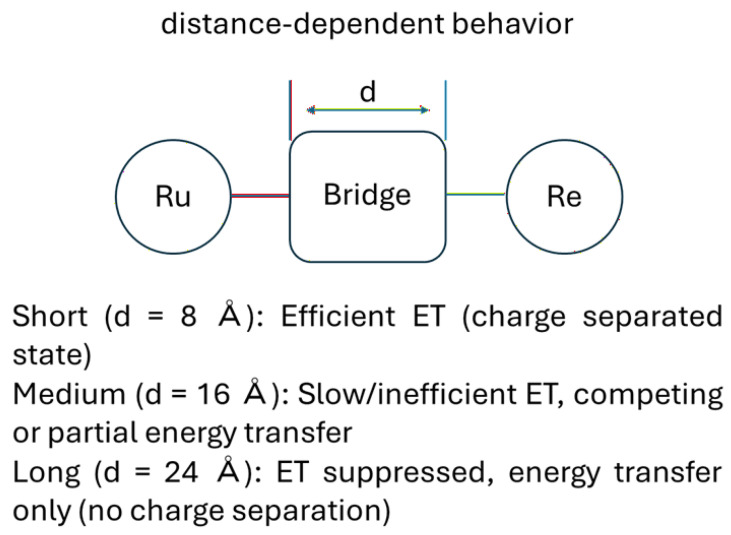
Distance- and matrix-dependent photophysical pathways in rigidly bridged Ru(II)–Rh(III) complexes [33]. In a rigid 77 K glass, all complexes exclusively underwent efficient Rh-to-Ru EnT as the electron-transfer pathways were completely frozen. In fluid solutions at 150 K, Ru-to-Rh forward electron transfer was selectively activated only in the shortest derivative lacking a phenyl spacer. This distance-dependent selectivity is governed by enhanced electronic coupling and minimized reorganization energy optimized within the Marcus normal region.

Photoinduced intramolecular pathways in rigidly bridged Ru(II)-Rh(III) complexes exhibit sharp selectivities between electrons and EnT. These depend strictly on matrix rigidity and intermetal distance. Indelli and co-workers systematically probed these effects by preparing a series of supramolecular assemblies with phenyl spacers that varied the metal-to-metal distance from 11 to 20 Å [33]. In a rigid 77 K glass, all complexes underwent efficient Rh-to-Ru EnT, as the electron-transfer quenching of the Ru excited state was completely frozen. In fluid solutions at 150 K, while the EnT was well maintained across all derivatives, Ru-to-Rh forward electron transfer was selectively activated only in the shortest complex (no phenyl spacers). This distance-dependent behavior arises because the compact geometry provides an excellent electronic coupling factor and small reorganization energy, optimizing the process within the thermally activated Marcus normal region (Figure 4).

### 2.2. CO_2_ Reaction with Complexes

In response to contemporary social demands and the need to balance environmental chemistry with efficient resource use, the reduction and utilization of carbon dioxide are being actively researched. In this context, when attempting to achieve catalytic reactions using photocatalytic systems, Ru and Rh complexes are useful light-absorbing components.

Non-covalent hydrogen bonding between a Ru(II) photosensitizer and a Re(I) catalyst significantly boosts photocatalytic CO_2_ reduction [36]. Cheung et al. developed a bimolecular system utilizing amide-functionalized Ru complexes. This system achieved a CO turnover number of 100 and a 23.3% quantum yield under 470 nm light, outperforming control systems that lacked amide groups. Mechanistically, the light-excited Ru complex undergoes reductive quenching via ligands to form the Ru complex. Despite a low driving force due to identical reduction potentials, hydrogen bonding drives the formation of a heterodimer. This supramolecular assembly accelerates inter-metal electron transfer, enabling efficient CO_2_ disproportionation into CO and carbonate without additive protons. Conversely, using N,N-Dimethylformamide (DMF) as a solvent disrupts these hydrogen bonds, markedly decreasing catalytic activity.

The combination of a Ru(II) carbonyl catalyst and a Ru(II) or Rh(II) polypyridyl photosensitizer drives the efficient photoreduction of CO_2_ to formate [37]. Ishida et al. constructed this selective system with the photosensitizer and triethanolamine as both the electron donor and proton source in DMF. Upon light irradiation at 320 nm, the excited photosensitizer is reductively quenched by this amine, yielding [Ru(bpy)_3_]^+^. Because the reduction potential of [Ru(bpy)_3_]^+^ (−1.35 V vs. Saturated Calomel Electrode (SCE)) is sufficiently more negative than that of the catalyst (−0.95 V vs. SCE), smooth PET occurs. The reduced catalyst then converts CO_2_ into formate, utilizing protons from this amine. ^13^C-NMR spectroscopy confirmed that the formate carbon originates exclusively from CO_2_, proving the photosensitizer’s pure, stable photofunctional role.

The luminescent excited state of [Ru(bpy)_3_]^2+^ undergoes rapid, diffusion-controlled electron-transfer quenching via electron-deficient organic molecules and transition metal ions. Bock et al. [38] used a flash photolysis relaxation technique to demonstrate this process. Light irradiation generates the long-lived excited state [Ru(bpy)_3_]^2+^. Introducing electron acceptors promotes a fast single-electron transfer. This direct quenching process yields a 1:1 ratio of the oxidized species [Ru(bpy)_3_]^3+^ and the reduced quencher. Subsequently, an ultra-fast thermal back-electron-transfer dark reaction returns the system to its ground state. Experiments revealed that this electron transfer pathway operates two orders of magnitude more effectively than competitive EnT routes [38].

**Figure 5:**
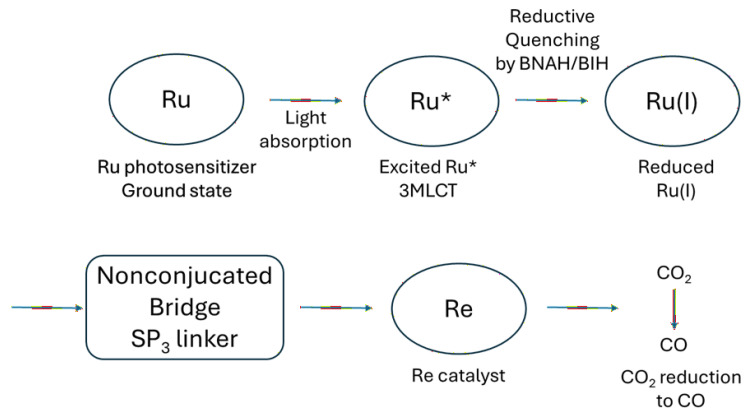
Photocatalytic CO_2_ reduction driven by non-covalent hydrogen bonding between a Ru(II) photosensitizer and a Re(I) catalyst [36]. Amide-functionalized Ru complexes form a supramolecular heterodimer via hydrogen bonding, which accelerates inter-metal electron transfer even under a low driving force. This assembly enables efficient CO_2_ reduction to CO and carbonate with high quantum yield and turnover numbers without external proton sources.

High-pressure and supercritical CO_2_ fluids significantly shift the product selectivity toward CO_2_ during photochemical CO_2_ reduction. Voyame et al. [39] evaluated this effect using a [Ru(bpy)_2_(CO)L]^n+^ catalyst paired with a [Ru(bpy)_3_]^2+^ photosensitizer in a biphasic water–DMF solution. Visible-light irradiation drives a compound to reductively quench the excited photosensitizer, generating [Ru(bpy)_3_]^3+^, which facilitates electron transfer. As a result, an intermediate is yielded. Increasing CO_2_ pressure from 10 to 150 bar accelerates the electrophilic attack of CO_2_ on this intermediate, boosting CO production. Conversely, formate production remains constant because the initial electron-transfer step is rate-limiting. The CO_2_ phase efficiently extracts the generated CO in order to prevent catalyst poisoning (Figure 5).

Triethanolamine is more than a simple base. It actively drives the catalytic cycle during photochemical CO_2_ reduction with Ru carbonyl complexes. Sampaio et al. utilized time-resolved infrared spectroscopy to investigate these dynamics with complexes containing 4,4′-dimethyl-2,2′-bipyridyl [40]. Following reductive quenching of the excited photosensitizer, a single-electron reduced catalyst is generated. This amine spontaneously captures CO_2_ to form a zwitterionic alkylcarbonate adduct. The adduct spatially assists hydride transfer from the metal hydride species to accelerate the reaction rate, and hydrogen bonding interactions facilitate the rapid release of the formate product.

Dynamic coordinative interactions between an Ir(III) photosensitizer and a Co catalyst increase quantum efficiency via an inner-sphere electron transfer mechanism. Wang et al. [41] developed this supramolecular assembly strategy using a pyridine-pendant Ir photosensitizer and Co phthalocyanine. Light irradiation drives the reductive quenching of the complex, generating a long-lived, single-electron-reduced species. The pendant pyridine group then reversibly coordinates to the axial position of the metal center, forming a 1:1 heteroassembly. This enables fast inner-sphere electron transfer. The approach achieved a 10.2% quantum yield for CO_2_-to-CO conversion, which was further optimized to 27.9% using an amino-functionalized catalyst to strengthen the coordinative bond. Crucially, the highly dynamic nature of this coordination prevents the blocking of active catalytic sites, ensuring efficient CO_2_ binding and turnover.

Overall, these examples demonstrate that efficient photocatalytic CO_2_ reduction requires a multifaceted approach to molecular design that extends beyond adjusting redox potential. Key structural factors include the control of reaction kinetics and the stabilization of intermediates. However, challenges remain regarding mass transport limitations in heterogeneous systems and the assurance of long-term photostability under high-pressure or extreme chemical conditions.

### 2.3. DSSC Application

Ru complexes are used as dyes in DSSCs. While there is fierce competition between chemical modifications of ligands, oxidation-reduction in the photoexcited state of the Ru complex is the most significant. It serves as the starting point for photoelectron transfer between the complex and the semiconductor that receives electrons.

Amphiphilic heteroleptic Ru(II) polypyridyl complexes bearing long aliphatic alkyl chains enable efficient long-term water stability in DSSCs. Murali et al. [42] developed three novel sensitizers featuring hydrophobic chains that assembled on nanocrystalline TiO_2_ thin films. Visible-light irradiation triggers a metal-to-ligand charge transfer to the π* orbital of the hydrophilic carboxylated ligand, driving efficient electron injection into the TiO_2_ conduction band with an incident photon-to-current conversion efficiency of up to 80%. Crucially, the long alkyl chains form a lateral hydrophobic network on the TiO_2_ surface.

Heteroleptic Ru(II) complexes functionalized with bulky cholesterol or long-chain alcohol units suppress dye desorption and charge recombination in solar cells. Klein et al. [43] reported a series of amphiphilic sensitizers, among which the complex ([Ru(II)LL^4^(NCS)_2_] (where L = 4,4‘-bis(carboxylic acid)-2,2‘-bipyridine)) achieved an outstanding photoelectric conversion efficiency of 8.8% under AM 1.5 sunlight. Visible-light absorption induces ultra-fast electron injection from the MLCT excited state into the nanocrystalline TiO_2_ conduction band, followed by rapid dye regeneration via the iodide/triiodide redox couple. The bulky hydrophobic groups mitigate electrostatic repulsion. They increase dye loading on the semiconductor surface and form a dense protective layer that physically prevents water-induced desorption and unwanted back-electron transfer.

A novel trithiocyanato-ruthenium(II) terpyridyl complex achieves efficient panchromatic sensitization from the visible-light range to the near-infrared region by 920 nm. Nazeeruddin et al. [44] designed this black dye system by tuning electronic effects to significantly red-shift the MLCT absorption. Three electron-donating thiocyanate ligands raise the energy of the Ru t_2g_ orbitals, while three carboxyl groups on the terpyridyl ligand simultaneously lower the π* energy levels. This concerted effect enables efficient PET from the excited state to the conduction band of nanocrystalline TiO_2_ films under broad-light-spectrum absorption. The resulting solar cell demonstrates a high incident-photon-to-current conversion efficiency of approximately 80% and a large photocurrent density of 20 mA/cm^2^.

DSSCs based on low-cost colloidal titanium dioxide thin films achieve high energy conversion efficiencies by separating light absorption from charge transport. O’Regan et al. [45] pioneered this system using a trinuclear Ru complex monolayer adsorbed onto a porous TiO_2_ matrix to harvest solar energy. Upon excitation, ultra-fast PET injects electrons from the dye into the TiO_2_ conduction band at a rate exceeding 10 to the 12th power per second. The oxidized dye is then rapidly regenerated back to its ground state by accepting electrons from an iodide/triiodide redox couple in solution. This minimized charge recombination yields a breakthrough conversion efficiency of up to 7.9% under simulated sunlight and 12% under diffuse light, maintaining exceptional photochemical stability over 5 million turnovers.

The cis-dithiocyanato Ru(II) complex, known as the N3 dye, achieves remarkable solar energy conversion by enabling ultra-fast electron injection and suppressed charge recombination. Nazeeruddin et al. [46] evaluated a series of Ru charge-transfer sensitizers on nanocrystalline TiO_2_ electrodes. Strong electronic coupling between the ligand π* orbitals and the TiO_2_ conduction band, mediated by robust carboxylate linkages, enables photoinduced electron injection within less than 7 ps at a near 100% quantum yield. The resulting oxidized Ru(III) species is rapidly regenerated by iodide on a nanosecond scale. Furthermore, 4-tert-butylpyridine is added to block active surface sites. As a result, dark current loss is caused by back-electron transfer being suppressed, boosting the open-circuit voltage and achieving a breakthrough 10% solar conversion efficiency.

DSSCs operate via a continuous electrochemical cycle where light absorption, ultrafast electron injection, and electrolyte-mediated dye regeneration are systematically synchronized. Sharma et al. [47] published a comprehensive review detailing the fundamentals, efficiency bottlenecks, and material advancements in these systems. Upon solar harvesting, the photosensitizer adsorbed on a wide-bandgap semiconductor undergoes an MLCT transition and enters an excited state. This excited electron is injected into the semiconductor conduction band and diffuses through the nanoparticle network to the external circuit. Finally, the oxidized sensitizer is returned to its ground state by accepting electrons from a redox couple reduced at the counter electrode and establishing a closed-loop system optimized by precise molecular design to minimize recombination losses.

Heteroleptic Ru complexes with extended π-conjugated ancillary ligands enhance the light-harvesting capability and open-circuit voltage of DSSCs by expanding their molar extinction coefficients. Gao et al. [48] developed two novel sensitizers that underwent MLCT excitation upon solar harvesting. Then, electrons were injected into the conduction band of nanocrystalline TiO_2_ thin films. The resulting oxidized complexes are rapidly returned to the ground state by iodide ions in the electrolyte. Due to its exceptionally high molar extinction coefficient of 17.5 multiplied by 10 to the 3rd power M^−1^cm^−1^, the carboxylated sensitizer maintains strong optical absorption even on thinner TiO_2_ films. This reduced film thickness suppresses dark current, yielding an outstanding solar conversion efficiency of up to 11.3% along with excellent long-term photothermal stability.

Bach et al. [49] pioneered this configuration by combining a standard Ru complex sensitizer with the first efficient solid-state DSSC, achieving a breakthrough monochromatic photon-to-electron conversion efficiency of 33%.

The incorporation of vinyl groups into bipyridine ligands expands the π-conjugated network of Ru(II) complexes to shift their absorption profile and maximize solar light harvesting. Klein et al. [50] synthesized a novel sensitizer, designated as the K8 complex, which exhibits a 20 nm red-shift in its MLCT absorption band and a 30% increase in its molar extinction coefficient compared to the standard N3 dye. Upon visible-light illumination, PET from the excited state injects electrons efficiently into the nanocrystalline TiO_2_ conduction band, driven by a favorable thermodynamic potential of minus 0.89 V vs. SCE. The resulting oxidized cation species has a slow charge recombination half-life of 200 μs with the injected electrons. This powerful optical absorptivity enables the use of thinner semiconductor films to suppress charge transport losses, achieving an outstanding solar conversion efficiency of 8.64% under simulated AM 1.5 sunlight.

Extending the bipyridine conjugation with alkoxystyryl groups yields heteroleptic Ru(II) complexes that overcome mass transport and charge losses in viscous electrolyte systems. Kuang et al. [51] developed new sensitizers, which undergo MLCT transitions and generate an excited state upon exposure to visible light. Ultrafast photoinduced electron injection into the porous TiO_2_ conduction band is completed within 20 fs, while the subsequent charge recombination with the oxidized dye is severely retarded to a half-life of 800 μs, allowing efficient dye regeneration via iodide. The high molar extinction coefficients of these complexes enable a significantly thinned photoanode layer of approximately 6.8 μm.

These examples demonstrate that, in addition to broadening the absorption wavelength, controlling molecular orientation at the semiconductor interface and film structure is crucial for enhancing DSSC efficiency. Strategies such as rendering ligands hydrophobic and extending π-conjugated systems increase adsorption density while effectively suppressing charge recombination and dye desorption. From a mechanistic standpoint, the synchronization of ultrafast electron injection and rapid dye regeneration governs performance. However, challenges remain regarding the assurance of long-term photothermal stability and the optimization of charge transport efficiency in solid-state systems; thus, a molecular design that simultaneously addresses these factors is a key objective for future research.

## 3. Working Hypothesis of PET Systems

If the light absorption wavelength, excited electronic state, excitation lifetime, redox potential, and photosensitization mechanism required for photoelectron transfer are not appropriate, the desired function cannot be obtained. Here, we deliberately present examples of working hypotheses, as well as trial and error scenarios, based on inappropriate molecular design guidelines “by the authors’ group in the past.”

As seen in previous sections, the general guidelines for material design can be broadly summarized as follows: Photoelectron transfer from metal complexes begins with a complex (such as Ru) that generates an excited triplet state due to light absorption at available wavelengths. The lifetime and quantum yield of the excited triplet state affect the efficiency of the photoelectron transfer reaction. However, the redox potential of the photoexcited species and the electron-receiving chemical species (a semiconductor such as TiO_2_ in the case of DSSC, or a complex catalyst in the case of a CO_2_ reduction catalyst) must be such that electron transfer is possible. Since electron transfer proceeds via chemical bonds, adsorption groups or conjugated bridging ligands are required.

One of the authors (T.A.) had experience verifying elemental technologies based on working hypotheses. Examples include light absorption of metal complexes and electron reactions of semiconductors based on simpler mechanisms. They used a salen-type metal complex containing an inexpensive first transition metal(II) ion. The light absorption wavelength and redox potential were adjusted with electron-withdrawing groups in the ligand or metal ions. Azo dyes were incorporated into the ligand to control the light absorption intensity. By chemically adsorbing carboxyl groups onto the semiconductor surface, the wavelength range of light during which the semiconductor undergoes electronic reactions could be altered (this mechanism would have been groundbreaking if realized). Fluorescence lifetime is controlled by the ligand substituents and central metal (diamagnetic or paramagnetic) through surface adsorption of silver nanoparticles.

### 3.1. The Adjustment of Light Absorption and Oxidation-Reduction Potential (Substituent Effects and Type of Metal) Is Not Appropriate

The introduction of electron-withdrawing substituents into chiral salen-type Cu(II) complexes effectively red-shifts their absorption profiles and optimizes their redox potential for enhanced solar cell performance. Shoji et al. [52] evaluated these modifications using time-dependent density functional theory and cyclic voltammetry. They found that electron-withdrawing groups boost peak absorption intensity and align orbital energy levels. In DSSCs, the light-harvesting complex reaches an excited state, allowing smooth PET to occur in the TiO_2_ conduction band because the reduction potential sits below −0.500 V. Simultaneously, an oxidation potential above +0.200 V ensures rapid dye regeneration by the electrolyte redox mediator, which is necessary to maintain a continuous photoelectrochemical cycle. Notably, a prototype device utilizing the optimized complex with the maximum absorption outperformed a standard system fabricated with the common Ru(II) dye N3.

Bromine-containing Schiff base Cu(II) complexes with extended π-conjugated ligands function as dual-purpose materials. They simultaneously serve as solar cell sensitizers and polymer flame retardants. Takahashi et al. [53] synthesized three such complexes, demonstrating that incorporating more bromine atoms systematically red-shifts the intense π-to-π* absorption bands up to 272 nm. According to theoretical design guidelines, these electron-withdrawing substituents expand light-harvesting capability by stabilizing the LUMO band. When deposited onto TiO_2_-based photoanodes in a DSSC, the complex absorbs incident photons until they reach an excited state, rapidly injecting an electron into the semiconductor conduction band to generate electricity before undergoing electrolyte-mediated reduction. Beyond their photoelectrochemical utility, highly brominated structures serve as efficient flame retardants, physically enhancing the incombustibility of host polymers like polymethyl methacrylate.

**Figure 6:**
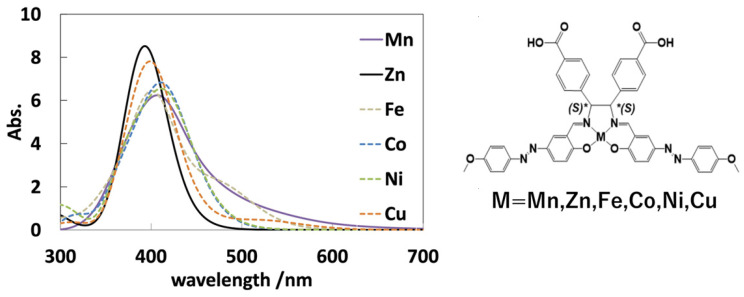
Experimental and calculated UV-Vis spectra and TD-DFT molecular orbital distributions of the divalent metal complexes. The complexes, characterized by various spectroscopic techniques and X-ray diffraction, exhibit broad UV-Vis absorption optimized by the π-conjugated naphthyl ring. TD-DFT calculations were performed to simulate the UV-Vis spectra and analyze the excited-state orbital distributions. The results reveal that the excited-state orbitals extend toward the -COOH anchoring site, ensuring strong electronic coupling for efficient electron injection into the TiO_2_ conduction band.

Yamaguchi et al. developed naphthyl-salen Schiff base complexes [ML(CH_3_OH)] (M = Mn, Fe, Co, Ni, Cu, Zn) with carboxyl groups to enhance dye adsorption on TiO_2_ for DSSC [54]. Characterized by elemental analysis, X-ray diffraction (XRD), infrared, and Ultraviolet–Visible (UV-Vis) near-infrared spectroscopy, these complexes exhibit light absorption optimized by the π-conjugated naphthyl ring. Time-dependent density-functional theory (TD-DFT) calculations revealed that efficient electron injection into the TiO_2_ conduction band requires the excited-state orbital distribution to extend toward the -COOH anchoring site to ensure strong electronic coupling. Unfavorable spatial distribution of these orbitals impairs electron transfer, significantly lowering the overall photovoltaic output of DSSC devices (Figure 6).

**Figure 7:**
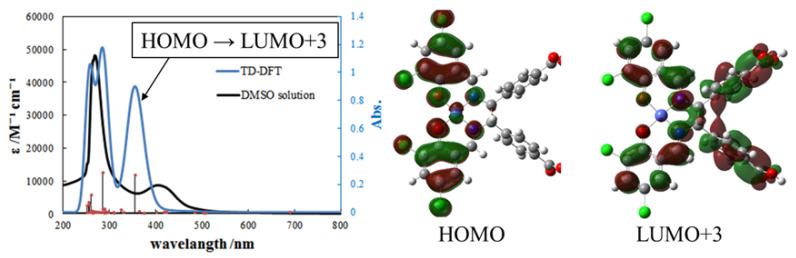
Experimental and DFT-calculated UV-vis spectra and orbital distributions HOMO and LUMO+3 for a salen metal complex. Complexes containing carboxyl groups were developed, and their successful chemisorption onto TiO_2_ surfaces was confirmed experimentally. TD-DFT calculations and predicted transitions demonstrate that these substituents significantly alter electric transition dipole moments, thereby optimizing the light-harvesting capacity.

Yamane et al. developed five chiral diphenyl-salen transition metal complexes (M = Fe(II), Co(II), Ni(II), Cu(II), Zn(II)) bearing carboxyl groups as novel dyes for DSSC [55]. X-Ray Photoelectron Spectroscopy (XPS) measurements confirmed the successful chemisorption of these complexes onto TiO_2_ surfaces. Incorporating electron-withdrawing groups induced a red shift in UV-Vis absorption spectra and significantly influenced the electric transition dipole moments calculated by TD-DFT, thereby optimizing their light-harvesting capacity. Electrochemical and theoretical characterizations (Figure 7) verified the fact that all compounds possess suitable orbital alignment and redox properties for efficient electron injection into the TiO_2_ conduction band and subsequent electrolyte regeneration, enabling continuous photocurrent generation.

### 3.2. The Dye Adsorption Density and Orientation on the Semiconductor Surface Are Not Appropriate

Tanaka et al. integrated azobenzene moieties into chiral salen-type metal complexes [56] to increase absorbance, shift absorption bands into the visible region, and enable photo-controlled dye adsorption on TiO_2_ for DSSCs. Although pristine salen complexes exhibit weak UV absorption, the azo groups introduced undergo light-induced cis-trans photoisomerization that improves light harvesting. Most notably, polarized UV light irradiation effectively modulates the adsorption density and orientation of the dyes on the TiO_2_ surface. This unique photo-control directly influences electronic coupling, facilitating energetically feasible electron injection into the semiconductor and tuning the final photovoltaic performance of the DSSC devices.

### 3.3. Spatial Distribution of Dipole Moments and Excitation Orbitals

Yamaguchi et al. utilized TD-DFT calculations to design six chiral salen Cu(II) complexes with extended π-conjugation and electron-withdrawing groups to shift DSSC light absorption toward the near-infrared region [57]. Both computational and experimental UV-Vis spectra confirmed a distinct bathochromic shift into the NIR band, successfully addressing the limited absorption window that often restricts the efficiency of standard DSSCs to 10% or less compared to silicon cells. Upon photoexcitation, the designed dye transfers an electron into the TiO_2_ conduction band to generate electrical output through the circuit, after which the oxidized dye is regenerated by the electrolyte redox couple. This work ultimately establishes a molecular design strategy to maximize light harvesting without requiring immediate device optimization.

Tsaturyan et al. evaluated binaphthyl-containing Schiff base Ni(II), Cu(II), and Zn(II) complexes with carboxyl groups as promising photosensitizers for DSSC [58]. Experimental data and (TD-)DFT modeling confirmed that their photovoltaic performance depends heavily on steric rigidity and adsorption geometry on TiO_2_ clusters. While all complexes exhibit similar adsorption energies through their anchoring carboxyl groups, the Zn(II) complex displays the highest molar extinction coefficient. This optimal electronic structure facilitates efficient light absorption and subsequent electron injection into the TiO_2_ conduction band, driving continuous power generation supported by electrolyte regeneration.

### 3.4. Excited State Lifetime (The Photoexcited State Cannot Be Properly Maintained)

**Figure 8:**
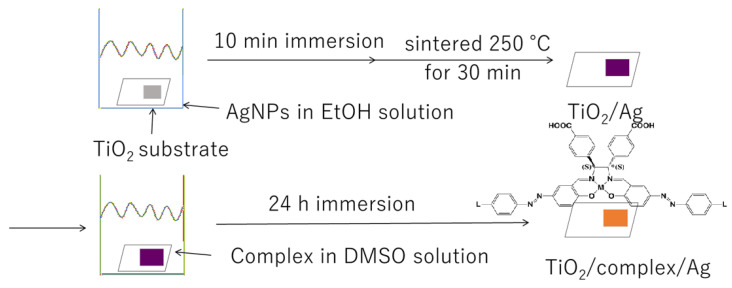
Preparation of DSSC systems in metal complexes TiO_2_ and AgNPs [59]. Time-resolved luminescence and transient absorption spectroscopy were utilized to elucidate the excited-state relaxation pathways of the plasmonic AgNP composite systems. Curve-fitting analysis of the streak camera and photon-counting data reveals that the composites possess longer fluorescence lifetimes than the corresponding sole metal complexes. These prolonged lifetimes indicate that the AgNP-integrated systems deactivate through three distinct reaction intermediates.

Saiga et al. designed chiral azo-salen Mn(II) and Zn(II) complexes to interact with silver nanoparticles (AgNPs) exhibiting localized surface plasmon resonance [59]. To elucidate the relaxation pathways from the excited state to the ground state, they performed time-resolved luminescence and transient absorption measurements using time-correlated single-photon counting and a streak camera. Curve-fitting analysis revealed that the plasmonic AgNP composite materials possessed longer fluorescence lifetimes than their corresponding sole metal complexes. This prolonged lifetime indicates that the composite systems relax through three distinct reaction intermediates during deactivation (Figure 8).

## 4. Conclusions and Perspective

The historical evolution of Ru tris-bipyridine complexes as photosensitizers highlights a growing focus on solvent-mediated cage-escape efficiencies rather than simple electron-transfer kinetics. Mechanistically, light absorption excites the Ru complex to a ^1^MLCT state, which undergoes rapid intersystem crossing into a long-lived triplet ^3^MLCT state. This triplet state is subsequently reductively quenched by an electron donor, generating free reduced species that actively deliver electrons to drive the target catalytic cycle.

Building upon these foundational principles, recent research points toward the use of supramolecular self-assembly to optimize local reaction environments and overcome diffusion limits. While traditional independent mixed-solvent systems experience weak spatial and orientation control, introducing dynamic non-covalent interactions can significantly accelerate interfacial electron transfer. To counteract water-induced viscosity changes that suppress cage-escape velocity, a hydrogen-bonded framework is proposed to preorganize Ru photosensitizers and Cu catalysts. This strategy effectively combines the operational simplicity of blended solutions with precise distance control, opening new avenues for highly efficient solar energy conversion.

As a future perspective, merging experimental approaches with theoretical calculations and data science will offer fascinating new insights into optimizing these PET systems. Integrating quantum chemical modeling with data-driven workflows allows researchers to efficiently screen spin–orbit coupling and vibronic factors, which are identified as pivotal in ultrafast nonadiabatic dynamics. By utilizing automated screening and machine learning, we can rapidly predict how photoinduced molecular vibrations alter potential energy surfaces to trigger specific conical intersections. This predictive power helps design metal complexes that rationally circumvent unproductive back-electron transfer, significantly extending the lifetime of critical charge-separated states for both photoreduction and photooxidation cycles. Furthermore, data science can accelerate the fine-tuning of ligand-to-metal charge transfer (LMCT) and mixed combinations of characteristics, guiding experiments toward targeted, selective single-electron abstraction. Ultimately, this synergistic loop between high-throughput computation, structural database analytics, and time-resolved spectroscopy transitions solar energy conversion from traditional trial-and-error discovery to predictable, highly efficient molecular design.

A comparative analysis of these failures is the core of this review. The following sections summarize the verification of elemental technologies, including the system (hypothesis) and results. The fact that traditional systems of photoelectron transfer involving metal complexes are yet to be surpassed is largely due to the lack of a material design that optimizes the overall system.

## Data Availability

No new data were created or analyzed in this study.

## Abbreviations

The following abbreviations are used in this manuscript:

DMF	*N*,*N*-Dimethylformamide
DSSC	Dye-Sensitized Solar Cell
EnT	Energy Transfer
LMCT	Ligand-to-Metal Charge Transfer
LUMO	Lowest Unoccupied Molecular Orbital
MLCT	Metal-to-Ligand Charge Transfer
PET	Photoinduced Electron Transfer
SCE	Saturated Calomel Electrode
TD-DFT	Time-dependent density-functional theory
UV	Ultraviolet–visible
Vis	Visible
XPS	X-Ray Photoelectron Spectroscopy
XRD	X-ray Diffraction
